# Intelligence

**Published:** 1826-12

**Authors:** 


### INTELLIGENCE.

### MONTHLY REPORT OF PREVALENT DISEASES.

We have very little to say on this subject in our present Report. Fever continues prevalent to an extent somewhat greater than usual at this season; and no considerable alteration in its character has taken place since last month. The cold damp weather, which has prevailed for some weeks, has given rise to numerous catarrhal affections, and occasionally to inflammation of the air-passages of a more severe character. In one instance under our observation, a man, about forty, died of this disease at the end of six days: the mucous membrane, from the epiglottis downwards as far as it could be traced, was of a bright red colour; and in the larynx, sloughing, with effusion of blood, had taken place.

*November 24th.*

*Application of Lunar Caustic.*—We have been favoured, through Dr. M. Hall, with the following communication on this subject from Mr. Higginbottom, of Nottingham.

In prosecuting my inquiry into the curative effects of the lunar caustic, I have had occasion to apply it in cases of erysipelas of the face, phagedenic ulcer of the penis, and in the purulent ophthalmia of infants. I hope soon to send you these cases in detail. In the mean time, however, I wish to observe—

1. That the lunar caustic applied to the surface in erysipelas, so as to form an eschar to the extent of one inch in breadth, on each side of the boundary of the erysipelatous inflammation, has appeared to present a complete barrier to its further extension, and rapidly to subdue the inflammation itself.

2. That the caustic applied on each side of the boundary of phagedenic ulcers of the penis, has, in like manner, checked the inflammatory and ulcerative processes.

3. That, in the purulent ophthalmia of infants, the application of the lunar caustic over the tumid eyelid, has had an almost immediate effect in subduing the disease.

The power of the lunar caustic in many diseases is, I am persuaded, not at all known; and especially its influence, under certain circumstances, in subduing inflammation. The inquiry into its various applications becomes daily more extended.

To obviate all misconception in regard to the nature and utility of the application of the caustic in such cases as I have alluded to above, I would observe, that it requires to be made with great care, and according to certain rules; and that it does not then induce either pain or ulceration. In the first instance, indeed, it would appear to affect the cuticle only, although its curative influence certainly *extends* to the subjacent textures. I would particularly caution my readers against applying the caustic too rashly. A severe application of the caustic is not what I recommend, and it might do harm, and so bring the remedy into undeserved disrepute. I hope to give a distinct account of the plan to be adopted in a succeeding Number.

---

*Ol. Terebinthinæ in Purpura.*—We have been favoured with the following note from Dr. Whitlock Nicholl:—

Sir,—In the last Number of the Medical and Physical Journal, two cases of Purpura are recorded. In the remarks which Dr. Hawkins has appended to his case of P. Hæmorrhagica, no notice is taken of the oleum terebinthinæ as a medicine "well calculated to answer the second indication" which he mentions, and as being a remedy "eminently useful in restraining the hemorrhage of purpura." I first employed the Ol. Tereb. in a case of P. Hæmorrhagica in 1818, and I communicated a statement of its salutary efficacy in that case to the Association of the Dublin College of Physicians. I have subsequently published cases illustrating its efficacy in restraining the hemorrhage of P. Hæmorrhagica, [London Med. Repos. July 1821, June 1824; Edinb. Med. Journal, October 1822;] and Mr. Thompson has published a case of P. Hæmorrhagica successfully treated by this remedy, [Repos. November 1823.] In the few cases of P. Hæmorrhagica which have fallen under my care, I have employed the oleum terebinthinæ with invariable success; I should, therefore, be glad to find that it had succeeded equally well in the hands of other practitioners.

I am, Sir, your obedient servant,

WHITLOCK NICHOLL.

12, *Old Burlington-street; Nov.* 4, 1826.

---

*Note on Vaccination.*

Sir,—Having observed a few queries in Dr. Gregory's paper on Vaccination, in your valuable Journal for November, which he wishes country practitioners to answer, I beg leave, for the Doctor's information, to state the result of my experience on the subject. I generally receive my supply of lymph from the National Vaccine Establishment, and find the *glasses* far more effectual than the *points*. The manner of using is as follows:—After slightly moistening the lymph on the glass with the smallest quantity of cold water, I make several incisions crossing each other, and then merely keep rubbing the lancet over the incisions for about a minute, paying particular attention to the Doctor's third particular,—viz. keeping the skin perfectly tense; a circumstance I have long found absolutely necessary in vaccinating. This I find by far the most effectual plan with dry lymph.

I have the honour to be, Sir, your obedient servant,

W. THOMAS, *Surgeon.*

*Pembroke Dock; 13th November*, 1826.

---

*Stammering.*

Sir,—Impediments of speech are so very distressing, and so many vain professions are every day made respecting their removal, that I consider it a duty which I owe to Dr. Hart, and to those afflicted with the above defect, to state that Dr. H. removed a very bad impediment of speech, of more than ten years' standing, in the case of one of my sons, and that in the course of a very few days. The process was conducted before my own eyes, and has its basis in strictly physiological principles.

I am, Sir, your most obedient servant,

JAMES JOHNSON, M.D.

*Suffolk-place, Haymarket; 20th November.*

---

*Edinburgh Journal of Medical Science.*—We have received a letter from the Editor of the Edinburgh Journal of Medical Science, and are perfectly satisfied with his explanation. We were not aware, at the time we noticed the mistake, that the proprietors of this Journal had written to him on the subject; otherwise we should not have noticed it. More importance has been given to the circumstance than it deserves. Now see vol. lvii. p. 189.

Sir Gilbert Blane.—We are much gratified to learn that this distinguished veteran has been elected a member of the Institute of France; a circumstance which may be regarded not only as an honour to that gentleman himself, but as highly complimentary to the medical profession in this country.

### METEOROLOGICAL JOURNAL,

*From October 20th, to November 20th, 1826.*

By Messrs. HARRIS and Co. Mathematical Instrument Makers, 50, High Holborn.

| October | Moon. | Rain gauge. | Thermom. | | | Barometer. | | De Luc's Hygrom. | | Winds. | | Atmospheric Variations. | | |
|---|---|---|---|---|---|---|---|---|---|---|---|---|---|---|
| | | | 9 A.M. | MAX. | MIN. | 9 A.M. | 10 P.M. | 9 A.M. | 10 P.M. | 9 A.M. | 10 P.M. | 9 *a.m.* | 2 *p.m.* | 10 *p.m.* |
| 20 | | | 60 | 62 | 58 | 29.85 | 29.85 | 96 | 95 | E | E | Cloudy | Fair | Cloudy |
| 21 | | | 59 | 69 | 58 | 29.85 | 29.88 | 96 | 89 | ESE | SSE | — | — | — |
| 22 | | .27 | 61 | 63 | 58 | 29.82 | 29.79 | 92 | 93 | S | SE | — | Rain | Rain |
| 23 | | .40 | 60 | 63 | 55 | 29.78 | 29.82 | 95 | 95 | SE | SW | Foggy | — | Starli. |
| 24 | ☾ | | 56 | 63 | 55 | 29.83 | 29.61 | 93 | 92 | WSW | SSW | — | — | — |
| 25 | | .18 | 58 | 58 | 45 | 29.35 | 29.35 | 87 | 82 | SW | W | Rain | — | Fair |
| 26 | | | 46 | 52 | 43 | 29.34 | 29.48 | 84 | 80 | WNW | WNW | — | Fair | Fine |
| 27 | | .23 | 47 | 53 | 40 | 29.54 | 29.70 | 84 | 78 | WSW | NW | — | Rain | — |
| 28 | | | 44 | 54 | 44 | 29.97 | 30.03 | 81 | 78 | WNW | W | Foggy | Fair | Foggy |
| 29 | | | 48 | 54 | 50 | 30.02 | 29.93 | 84 | 93 | WSW | W | — | Cloudy | Sm. Ra. |
| 30 | | .4 | 50 | 56 | 47 | 29.99 | 29.94 | 95 | 95 | NE | WSW | Rain | Rain | Foggy |
| 31 | ● | | 50 | 52 | 43 | 29.97 | 29.91 | 78 | 83 | NW | W | Cloudy | Fair | Sl. Rain |
| Nov. | | | | | | | | | | | | | | |
| 1 | | | 44 | 50 | 40 | 29.64 | 29.53 | 85 | 79 | SW | NNW | Foggy | — | Starli. |
| 2 | | | 42 | 50 | 42 | 29.67 | 29.76 | 83 | 87 | N | N | Fine | Fine | — |
| 3 | | .103 | 46 | 53 | 47 | 29.78 | 29.66 | 90 | 96 | N | NE | — | — | Rain |
| 4 | | | 48 | 48 | 42 | 29.64 | 29.77 | 98 | 97 | ENE | ENE | Rain | Rain | Fine |
| 5 | | | 46 | 50 | 44 | 29.75 | 29.69 | 97 | 94 | ENE | NNW | Cloudy | — | Rain |
| 6 | ☽ | .28 | 45 | 48 | 31 | 29.66 | 29.73 | 95 | 84 | WNW | N | — | — | Cloudy |
| 7 | | | 32 | 42 | 33 | 29.81 | 29.92 | 78 | 78 | N | WNW | Fogg | Fine | Fine |
| 8 | | | 37 | 41 | 38 | 29.97 | 30.00 | 78 | 78 | WNW | WNW | — | — | — |
| 9 | | | 35 | 43 | 32 | 30.03 | 30.10 | 84 | 83 | N | N | — | — | — |
| 10 | | | 35 | 48 | 46 | 29.97 | 29.78 | 85 | 96 | WSW | W | — | Sm. Ra. | Sm. Ra. |
| 11 | | | 48 | 53 | 43 | 29.68 | 29.55 | 97 | 94 | WSW | SW | — | — | Fine |
| 12 | | | 45 | 53 | 36 | 29.54 | 29.40 | 93 | 83 | SW | WSW | Cloudy | Cloudy | — |
| 13 | | .40 | 38 | 46 | 38 | 29.39 | 28.85 | 88 | 98 | WSW | ESE | — | Rain | Rain |
| 14 | ○ | | 39 | 46 | 38 | 28.98 | 29.14 | 91 | 78 | WNW | NW | — | Cloudy | Fine |
| 15 | Ec | | 40 | 46 | 33 | 29.46 | 29.67 | 82 | 80 | WNW | WNW | — | — | — |
| 16 | | | 35 | 44 | 43 | 29.84 | 29.86 | 90 | 80 | W | S | Foggy | — | Cloudy |
| 17 | | | 46 | 47 | 38 | 29.79 | 29.97 | 91 | 90 | SSE | E | Sm. Ra. | — | — |
| 18 | | .25 | 43 | 46 | 44 | 30.02 | 30.02 | 91 | 95 | NE | NE | — | — | Sm. Ra. |
| 19 | | | 45 | 47 | 43 | 30.04 | 30.11 | 88 | 98 | NE | NNEv | — | Sm. Ra. | — |

The quantity of Rain fallen in the month of October, was 1 inch and 90.100ths.

